# *Paraburkholderia sabiae* administration alters zebrafish anxiety-like behavior *via* gut microbial taurine metabolism

**DOI:** 10.3389/fmicb.2023.1079187

**Published:** 2023-02-16

**Authors:** Shunsuke Ichikawa, Reimi Abe, Haruka Fujimoto, Koushi Higashi, Liqing Zang, Hiroko Nakayama, Izumi Matsuoka, Yasuhito Shimada

**Affiliations:** ^1^Faculty of Education, Mie University, Tsu, Mie, Japan; ^2^Mie University Zebrafish Drug Screening Center, Tsu, Japan; ^3^Graduate School of Regional Innovation Studies, Mie University, Tsu, Mie, Japan; ^4^Department of Integrative Pharmacology, Mie University Graduate School of Medicine, Tsu, Japan; ^5^Department of Bioinformatics, Mie University Advanced Science Research Promotion Center, Tsu, Japan

**Keywords:** *Danio rerio*, Burkholderiaceae, novel tank diving, stress, mental diseases

## Abstract

Interventions to the gut microbiome manipulate the gut–brain axis and could be useful in the treatment of anxiety and depression. In this study, we demonstrated that administration of the bacterium *Paraburkholderia sabiae* reduces anxiety-like behavior in adult zebrafish. *P. sabiae* administration increased the diversity of the zebrafish gut microbiome. Linear discriminant analysis Effect Size (LEfSe) analysis revealed that the populations of Actinomycetales including Nocardiaceae, *Nocardia*, Gordoniaceae, *Gordonia*, Nakamurellaceae, and Aeromonadaceae were reduced, whereas those of Rhizobiales including Xanthobacteraceae, Bradyrhizobiaceae, Rhodospirillaceae, and Pirellulaceae were increased in the gut microbiome. Functional analysis using Phylogenetic Investigation of Communities by Reconstruction of Unobserved States (PICRUSt2) predicted that *P. sabiae* administration altered taurine metabolism in the zebrafish gut, and we demonstrated that *P. sabiae* administration increased the taurine concentration in the brain. Since taurine functions as an antidepressant neurotransmitter in vertebrates, our results suggest that *P. sabiae* could improve anxiety-like behavior in zebrafish *via* the gut-brain axis.

## 1. Introduction

The gut microbiome affects brain development, myelination patterns (Gacias et al., 2016; Hoban et al., 2016), and host behaviors (Vuong et al., 2017) by modulating neurotransmitter synthesis in the gut and brain (Needham et al., 2020; Huang and Wu, 2021). Metabolites produced by the gut microbiome are distributed throughout all organs, including the brain, thereby affecting neuronal cell development and behaviors (Swann et al., 2017). For example, *Lactobacillus rhamnosus* JB-1, *Lactobacillus helveticus* R0052, and *Bifidobacterium longum* R0175 reduce stress-induced corticosterone as well as anxiety- and depression-related behaviors in rodents (Bravo et al., 2011; Messaoudi et al., 2011). *Lactobacillus helveticus* R0052 and *Bifidobacterium longum* R0175 also alleviated psychological distress in human subjects (Messaoudi et al., 2011). Moreover, consumption of fermented milk containing *Bifidobacterium animalis* subsp. *lactis*, *Streptococcus thermophiles*, *Lactobacillus bulgaricus*, and *Lactococcus lactis* subsp. *lactis* altered the activity of brain regions that control the central processing of emotion and sensation in healthy women (Tillisch et al., 2013). In addition to probiotic studies, Needham et al. (2022) reported that a gut-derived molecule, 4-ethylphenyl sulfate, modulates anxiety-like behavior in mice by affecting oligodendrocyte function and myelin patterning in the mouse brain. These studies suggest the possibility that interventions to the gut microbiome manipulate the gut–brain axis and could be useful in the treatment of anxiety and depression.

Zebrafish (*Danio rerio*) is becoming a major animal model for neurobehavioral research. Zebrafish exhibit numerous behaviors that correlate with human neurological processes and disorders, such as anxiety (Li et al., 2015), learning (Andersson et al., 2015), fear (Colwill and Creton, 2011), sociability (Engeszer et al., 2007), and psychosis (Giacomini et al., 2006). The zebrafish neurotransmitter system is similar to that of other vertebrates, including rodents and humans (Rico et al., 2011). Several zebrafish neurobehavioral tests have been developed and validated using chemicals and natural compounds. The novel tank diving test, which is suitable for evaluating anxiety-like behavior, is commonly used for drug testing, chemical screening, and mechanism identification (Wong et al., 2013). Similar to humans, the gut microbiome also affects the normal neurobehavioral phenotypes in zebrafish (Stagaman et al., 2020). For example, impairment of microbial colonization in zebrafish larvae using antibiotics caused hyperactivity (Phelps et al., 2017). Dietary administration of *L. rhamnosus* IMC 501 affects brain-derived neurotrophic factor (BDNF) levels, serotonin metabolism, and shoaling behavior in zebrafish (Borrelli et al., 2016). *Lactobacillus plantarum* administration alters GABAergic and serotonergic signaling in the brain and reduces anxiety-like behavior in zebrafish (Davis et al., 2016).

In this study, we focused on the bacterium *Paraburkholderia*, whose functions in the human and rodent gut microbiome have been previously discussed (Haonon et al., 2021; Zhang et al., 2021; Ning et al., 2022). Among these, we identified that administration of *Paraburkholderia sabiae* reduced anxiety traits in zebrafish. *P. sabiae* administration increased the diversity of the zebrafish gut microbiome and altered the taurine metabolism in zebrafish.

## 2. Materials and methods

### 2.1. *Paraburkholderia sabiae* exposure to the zebrafish

Wild-type zebrafish (Danio rerio; AB strain, Zebrafish International Research Center, Eugene, OR, United States) were raised in a flow-through system at 28 ± 0.5°C under a 14/10 h light/dark cycle at the Zebrafish Drug Screening Center, Mie University, Japan. Ten AB wild type 6 months-old zebrafish were transferred to 2 L of rearing water at 28°C. The fish were fed at 9:00 a.m. and 5:00 p.m. on weekdays. For 1 month, *P. sabiae* cells (1.0 × 10^9^ cell/L) were administered in the rearing water twice a day at feeding times. *Paraburkholderia sabiae* was purchased from the German Collection of Microorganisms and details for the culture is described in Supplementary online materials. The experimental procedures were performed in accordance with the Japanese Animal Welfare Regulatory Practice Act on Welfare and Management of Animals (Ministry of the Environment of Japan) and in compliance with ARRIVE guidelines^1^. Ethical approval from the local Institutional Animal Care and Use Committee was not sought, because this law does not mandate the protection of zebrafish.

### 2.2. Evaluation of the anxiety-like behavior in zebrafish

Anxiety in zebrafish was evaluated using the novel tank diving test (Haghani et al., 2019; Anwer et al., 2021) from 14:00 p.m. to 15:30 p.m. An observation tank (149 mm × 38 mm × 100 mm) was filled with rearing water at a height of 10 cm. The zebrafish were transferred from the rearing tank to a restraint tank (37 mm × 7 mm × 65 mm) with a net. The zebrafish were placed in a restraint tank for 5 min to cause anxiety and transferred to the observation tank. When the zebrafish were transferred to the observation tank, their movements were immediately captured as videos using an iPad Pro for 2 min. The average speed, acceleration, total travel distance, and exploration rate in the observation tank were analyzed using the ToxTrac software (Rodriguez et al., 2018). Supplementary Figure S1 shows a schematic of the evaluation of zebrafish anxiety.

### 2.3. Gut microbiome analysis in zebrafish

DNA was extracted from the zebrafish intestine using a Quick-DNA Fecal/Soil Microbe Miniprep Kit (Zymo Research, Irvine, CA, United States). The 16S rRNA V3/V4 region in the DNA was amplified by PCR, and Library preparation and sequencing were performed using the MiSeq Reagent Kit v3 (Illumina, San Diego, CA, USA) at 2 × 300 bp. The microbiome data were analyzed by phylogenetic investigation of communities by reconstruction of unobserved states 2 (PICRUSt2) ver. 2.3.0 b 32 (Douglas et al., 2020). Linear discriminant analysis effect size (LEfSe; Segata et al., 2011). For details, please see the Supplementary online materials.

### 2.4. Measurement of taurine and serotonin concentrations in the zebrafish brains

Zebrafish brains were thoroughly homogenized using a pestle in 100 μl PBS cooled on ice. The supernatant was collected by centrifugation at 14,500 rpm for 2 min. One microliter of the 10-fold diluted supernatant was used to measure taurine and serotonin concentrations using the Taurine Assay Kit (Cell Biolabs, San Diego, CA, USA) and Serotonin ELISA Kit (Immusmol, Bordeaux, France), respectively, according to the manufacturers’ protocols.

## 3. Results

### 3.1. *Paraburkholderia sabiae* administration reduced anxiety-like behavior in zebrafish

**Figure 1:**
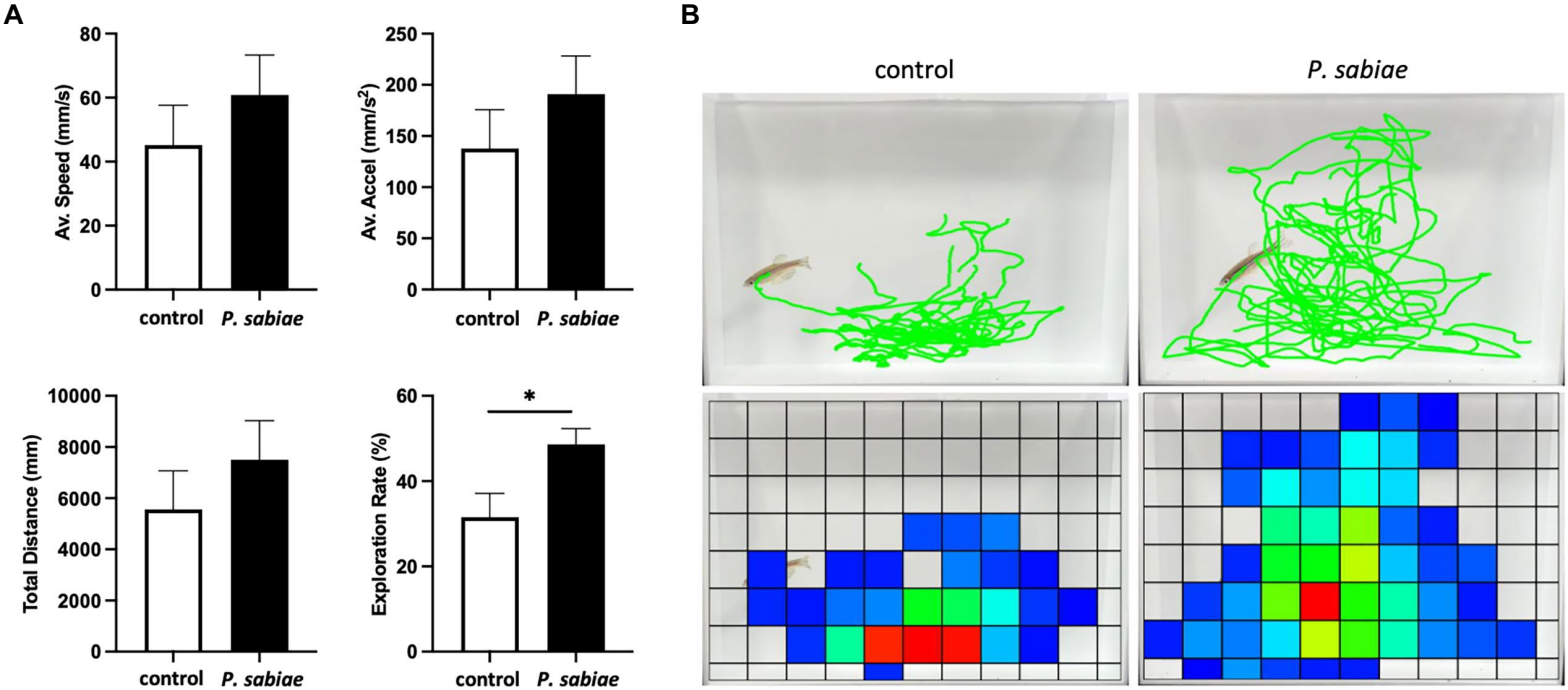
The zebrafish anxiety behaviors evaluated by the novel tank diving test. **(A)** Movements of the zebrafish following the transfer into the observation tank were traced for 2 min. The average speed (mm/s), average acceleration (mm/s^2^), total travel distance (mm), and exploration rate in the observation tank (%) of the zebrafish are presented. The error bars indicate standard error. *N* = 5, *p < 0.05. **(B)** The representative images of the traced movement of the *P. sabiae*-exposed and the control zebrafish.

Six months-old zebrafish were exposed to *P. sabiae* bacterial cells for 1 month, and their anxiety-like behavior was evaluated using a novel tank diving test, which has become a standard test for determining anxiety-like behavior in zebrafish (Haghani et al., 2019; Supplementary Figure S1; Supplementary movies S1, S2). The average speed, average acceleration, and total distance tended to be higher in *P. sabiae*-exposed zebrafish. The average speeds were 45.1 mm/s and 60.8 mm/s, the average accelerations were 137.8 mm/s^2^ and 190.0 mm/s^2^, and the total distances were 5,566 mm and 7,501 mm in the control and the *P. sabiae*-exposed zebrafish, respectively (Figure 1A). Furthermore, *P. sabiae*-exposed zebrafish migrated significantly more extensively in the observation tank. The exploration rate in the observation tank was 32% for the control zebrafish, whereas that for the *P. sabiae*-exposed zebrafish was 49% (*p* < 0.05; Figures 1A,B). There was no significant difference in exploration rate between the genders (Supplementary Figure S2). The response of adult zebrafish in the novel tank environment, that is, to initially stay at the bottom of the tank and eventually acclimating to the rest of the tank, is interpreted as a precautionary anti-predator response and anxiety reduction, respectively (Haghani et al., 2019). Our results indicated that *P. sabiae* administration reduced anxiety in zebrafish.

Multiple behaviors, including stress and anxiety, are modulated by neuropeptides (Rana et al., 2022). The altered expression of *bdnf* in distinct brain areas and BDNF signaling associated with social behavior have been frequently demonstrated in gut-brain axis models in mice (Bercik et al., 2010; Diaz Heijtz et al., 2011; Desbonnet et al., 2015). We also evaluated the expression levels of anxiety-related genes, neuropeptide Y *npy* (Shiozaki et al., 2020), isotocin (*oxtI*) (Godwin and Thompson, 2012), and *bdnf* (Bjorkholm and Monteggia, 2016). *oxti* expression tended to increase in the brains of *P. sabiae*-exposed fish, whereas *npy* expression decreased (no significance, Supplementary Figure S3). *bdnf* expression also tended to increase in the *P. sabiae*-exposed fish brain, similar to the results of a previous zebrafish study (Borrelli et al., 2016).

### 3.2. *Paraburkholderia sabiae* administration increased the microbial diversity in the zebrafish gut

**Figure 2:**
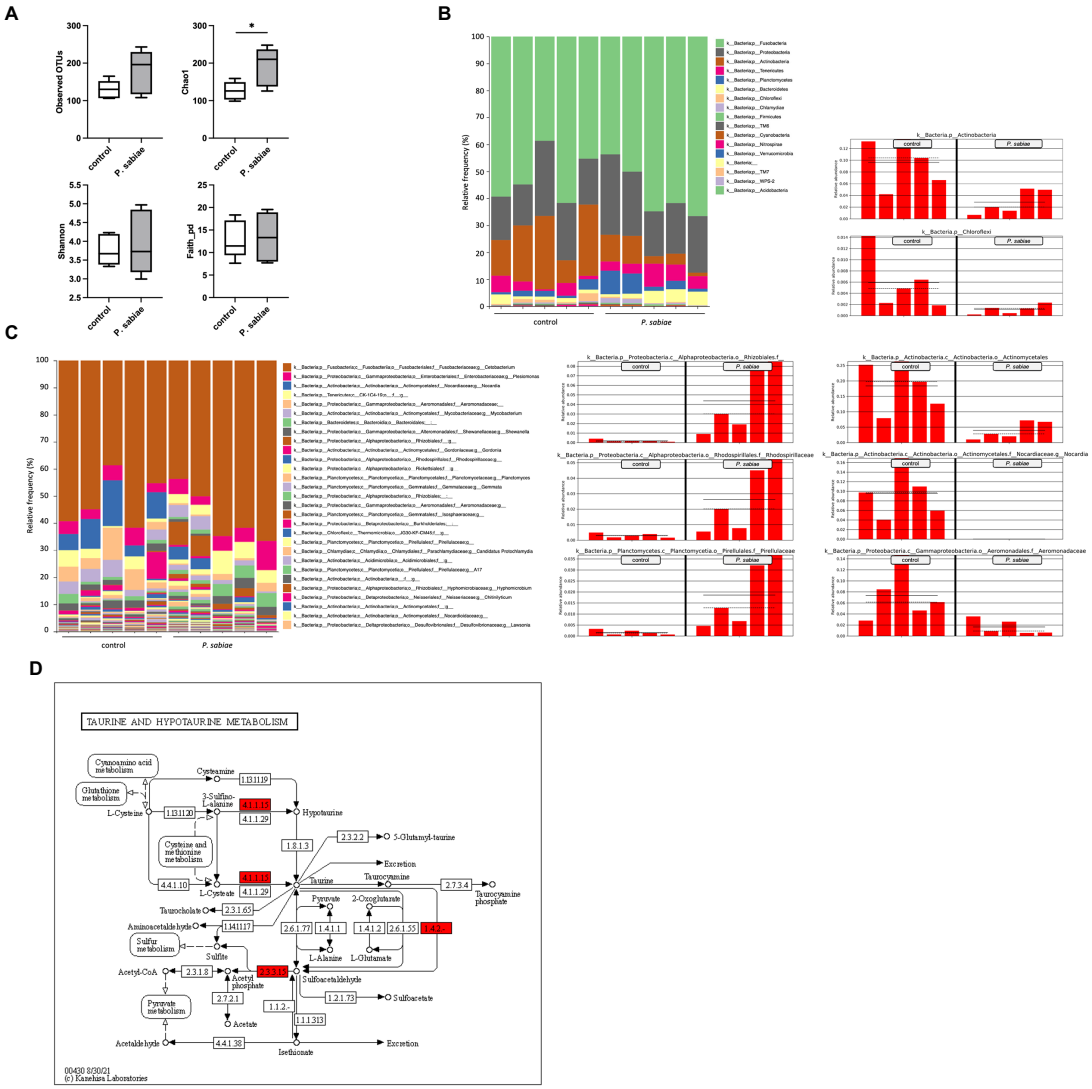
The gut microbiome analysis of the *P. sabiae*-exposed zebrafish. **(A)** The diversity indices, Observed OTUs, Chao1, and Shannon, Faith_pd in the *P. sabiae*-exposed and the control zebrafish gut microbiomes. All OTUs detected by the microbiome analysis are summarized in Supplementary Table S2. **(B)** The phylum level and **(C)** genus level analyses of the zebrafish gut microbiome were conducted. The representative OTUs which increased and decreased in the *P. sabiae*-exposed zebrafish gut microbiomes are extracted by the LEfSe analysis. All OTUs extracted by the LEfSe analysis are summarized in Supplementary Table S3. **(D)** The PICRUSt2 functional analysis with METACYC database revealed that the functions of superpathway of taurine degradation (PWY-1541) was significantly reduced in the *P. sabiae*-exposed zebrafish gut microbiome (Supplementary Table S7). The predicted taurine metabolism pathways are represented with KEGG Mapper. Reduced functions in the taurine metabolic pathway in the *P. sabiae*-exposed zebrafish gut microbiome are shown as red. *N* = 5, *p < 0.05.

The gut microbiomes of the five *P. sabiae*-exposed zebrafish were analyzed using the 16S rRNA V3/V4 region of their gut microbial DNAs. The 30,000–50,000 pair-reads, 93.1–94.1 Q20 values, and 83.5–85.3 Q30 values were obtained for each sample. A total of 450 OTUs were generated from the analysis using Qiime2 (Supplementary Table S2). Despite the continuous feeding of the bacterial cells to the zebrafish for 1 month, the observed OTUs in the *P. sabiae*-exposed zebrafish gut microbiome tended to be higher than those in the control zebrafish. Chao1, the richness index of alpha diversity in the gut microbiome, was significantly higher in the *P. sabiae*-exposed zebrafish than in the control zebrafish (*p* < 0.05; Figure 2A). No significant difference in the evenness index of the Shannon diversity index and the indicator of phylogenetic diversity, Faith_pd, was observed between these zebrafish gut microbiomes. *P. sabiae* was not detected in the gut microbiome analysis (Supplementary Table S2); thus, *P. sabiae* functioned without colonizing the zebrafish gut.

LEfSe analysis was used to compare the bacterial populations between the *P. sabiae*-exposed and control zebrafish gut microbiomes (Supplementary Table S3; Segata et al., 2011). At the phylum level, Fusobacteria and Proteobacteria were dominant in the zebrafish gut microbiome, which is consistent with previous reports (Borrelli et al., 2016; Davis et al., 2016). Actinobacteria and Chloroflexi populations were smaller in the gut microbiome of *P. sabiae*-exposed zebrafish than in that of control zebrafish (Figure 2B). At the genus level, *Cetobacterium* was detected as the major bacterium in the gut microbiome of zebrafish. The populations of Actinomycetales, including Nocardiaceae (*p* < 0.01), *Nocardia* (*p* < 0.01), Gordoniaceae (*p* < 0.01), *Gordonia* (*p* < 0.01), Nakamurellaceae (*p* < 0.01), and Aeromonadaceae (*p* < 0.05) were significantly reduced, whereas those of Rhizobiales, including Xanthobacteraceae (*p* < 0.05) and Bradyrhizobiaceae (*p* < 0.05), Rhodospirillaceae (*p* < 0.01), and Pirellulaceae (*p* < 0.01) were significantly increased in *the P. sabiae* group (Figure 2C; Supplementary Table S3).

Functional composition tables based on EC number, Kyoto Encyclopedia of Genes and Genomes (KEGG) ortholog, cluster of orthologous groups, and METACYC were generated using PICRUSt2 (Supplementary Tables S4–S7; Douglas et al., 2020). Functional analysis based on METACYC revealed that the mevalonate pathway (PWY-6174), superpathway of polyamine biosynthesis (PWY-6565), and chitin derivative degradation (PWY-6906) were detected only in the *P. sabiae*-exposed zebrafish gut microbiome. In contrast, the functions of the superpathways of taurine degradation (PWY-1541) (*p* < 0.01), creatinine degradation (PWY-4722) (*p* < 0.01), and aerobic toluene degradation *via* catechol (PWY-5178) (*p* < 0.001) were significantly reduced in the *P. sabiae*-exposed zebrafish gut microbiome (Supplementary Table S7). Regarding the neurotransmitter taurine, glutamate decarboxylase [EC:4.1.1.15] (*p* < 0.01; Supplementary Table S4) and the functions of *tauY*: taurine dehydrogenase large subunit [EC:1.4.2.-] (K07256) (*p* < 0.05), *tauA*: taurine transport system substrate-binding protein (K07256) (*p* < 0.05; Supplementary Table S5), ABC-type taurine transport system, periplasmic component (COG4521; *p* < 0.05), and ATPase component (COG4525) (*p* < 0.01; Supplementary Table S6) were also reduced in the *P. sabiae*-exposed zebrafish gut microbiome. The reduced functions in the metabolic pathways of taurine and hypotaurine were represented by the KEGG Mapper (Kanehisa et al., 2022; Figure 2D).

### 3.3. Effects of *Paraburkholderia sabiae* on taurine levels in the zebrafish brain

**Figure 3:**
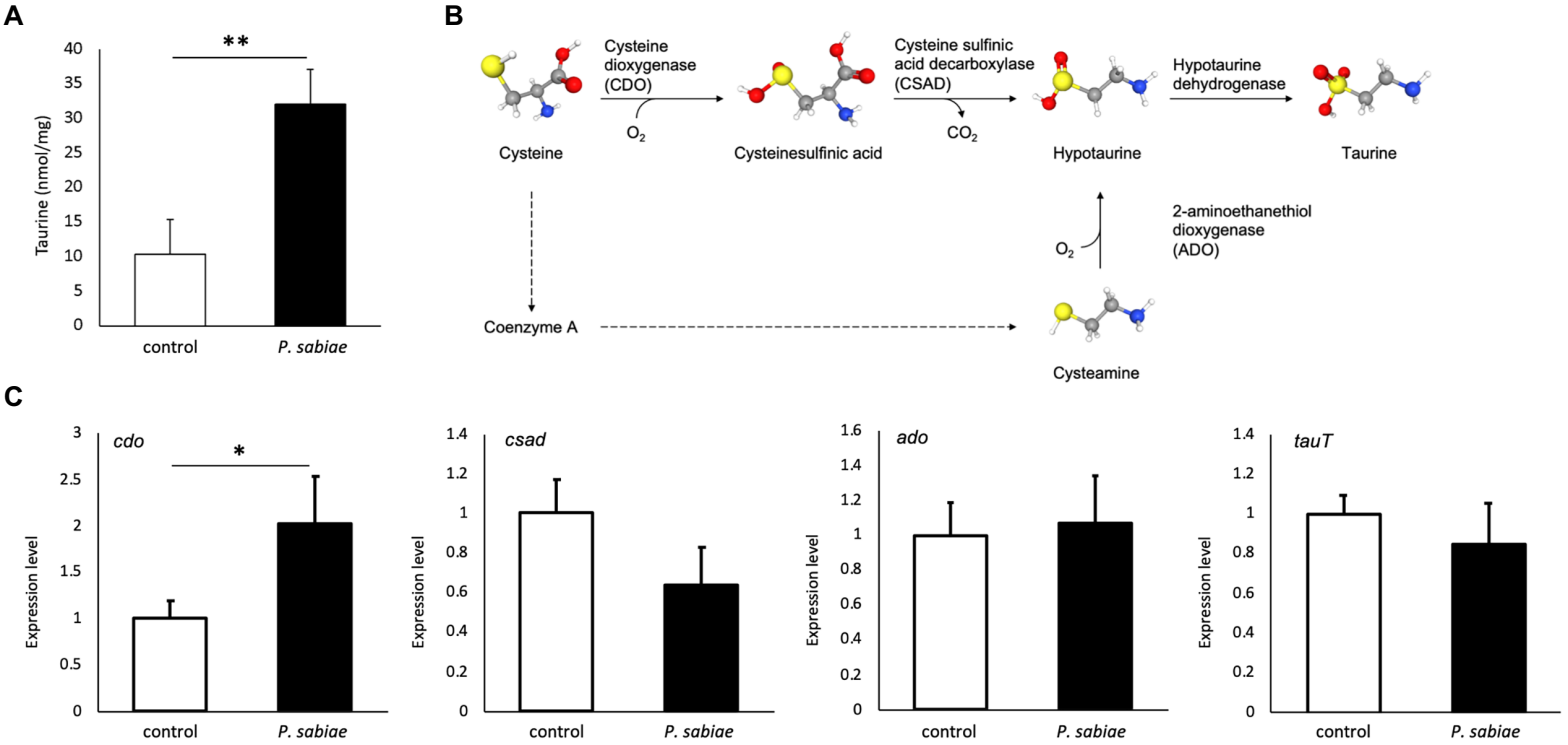
Taurine concentration and expression of taurine synthesis genes in zebrafish brains. **(A)** Taurine concentrations in zebrafish brains evaluated using the Taurine Assay Kit (Cell Biolabs.). *N* = 5, **p < 0.01. **(B)** Taurine biosynthesis pathways, and **(C)** the expression levels of taurine biosynthesis genes (*ado*, *cdo*, *csad*, *tauT*) in the zebrafish brain. *N* = 8, *p < 0.1.

Because PICRUSt2 predicted downregulation of the superpathway of taurine degradation (PWY-1541) in the zebrafish gut microbiome, we next evaluated taurine levels in zebrafish. The concentration of taurine (2-aminoethanesulfonic acid) in the *P. sabiae* group was significantly (*p* < 0.01) higher than that in the control zebrafish brain:32.2 and 10.4 nmol/mg-brain, respectively (Figure 3A). We also measured the taurine level of intestinal contents (microbiota) and blood, and found that *P. sabiae* administration shows a tendency (*p* < 0.2) to increase taurine in the intestinal contents (Supplementary Figure S4). The expression level of the taurine biosynthesis gene encoding cysteine dioxygenase *cdo*, was 2.0 times higher than that in the *P. sabiae*-exposed zebrafish brains, compared with that in the control zebrafish brains (*p* < 0.1), while no difference was observed in those of the other taurine biosynthesis genes encoding cysteamine dioxygenase *ado*, cysteine sulfinic acid decarboxylase *csad*, and taurine transporter *tauT* (Figures 3B,C).

In addition to taurine, serotonin (5-hydroxytryptamine) and GABA are also well-known key players in anxiety in human and zebrafish (Davis et al., 2016; Marcinkiewcz et al., 2016). Several probiotic treatments affect serotonin-related gene expressions in zebrafish (Borrelli et al., 2016; Davis et al., 2016). The expression level of the serotonergic gene *tph2* was significantly increased in the *P. sabiae* group, however that of the other serotonergic genes *slc4a6a*, *slc4a6b*, *htr1aa*, *mao*, *tph1a*, and *tph1b* were not significantly different between these zebrafish brains (Supplementary Figure S5). No significant difference of the serotonin concentrations was observed in these zebrafish brains: (6.0 and 6.2 pmol/mg-brain), according not to be predicted by PICRUSt2. For GABAergic genes, the expression levels of *gabra1* and *gad1* were not affected by *P. sabiae* administration (Supplementary Figure S6).

## 4. Discussion

### 4.1. The characteristics of *Paraburkholderia* in gut microbiome

*Paraburkholderia* is a new genus in gram-negative β-proteobacteria, which can be distinguished from *Burkholderia* (Sawana et al., 2014). *Paraburkholderia* is often isolated from the rhizosphere and plant organs, such as root nodules, and it functions in nitrogen fixation and plant growth promotion (Esmaeel et al., 2018). Furthermore, *Paraburkholderia* has been detected in guts, ranging from those of insects to humans (Takeshita and Kikuchi, 2020; Chen et al., 2022; Tang et al., 2022). The presence of *Burkholderia*-*Caballeronia*-*Paraburkholderia* in human gut microbiomes remarkably correlated with body mass index, osteoarthritis, and cholangiocarcinoma (Zhang et al., 2021; Ning et al., 2022). *Opisthorchis viverrini* infection is a key risk factor for cholangiocarcinoma and increases *Paraburkholderia* in the gut microbiomes of hamsters (Haonon et al., 2021). The abundance of *Paraburkholderia* has also been discussed with regards to ulcerative colitis (Liu et al., 2021) and breast cancer-related fatigue (Lv et al., 2022).

In this study, we focused on the function of *Paraburkholderia* and demonstrated that zebrafish exposed to the *Paraburkholderia* type strain, *P. sabiae* DSM 23623 (Chen et al., 2008), moved more extensively in the novel tank diving test (Figure 1), suggesting the possibility that the *P. sabiae*-exposed treatment reduced anxiety in zebrafish. This is the first report to describe the function of *Paraburkholderia* in animal behavior.

### 4.2. *Paraburkholderia sabiae* administration affected the diversity of zebrafish gut microbiome

A loss in the diversity of the gut microbiome and dysbiosis caused by diet, infection, stress, etc., leads to abnormalities in brain function and mental health (Rogers et al., 2016). Antibiotic dysbiosis is also associated with an elevated risk of anxiety (Lurie et al., 2015). Borrelli et al. (2016) and Davis et al. (2016) reported that bacterial administration modulates gut microbial communities in zebrafish. Here, we show that *P. sabiae* administration increased the richness of zebrafish gut microbiomes, as shown by the high values of the diversity index, Chao1 (Figure 2A). In addition to the higher richness, we observed a reduction of Actinobacteria in the gut microbiome in the *P. sabiae*-exposed zebrafish, which represented reduced anxiety-like behavior (Figures 1, 2), which was similar to the case of *L. rhamnosus* administration (Borrelli et al., 2016). *Paraburkholderia* exposure treatment unexpectedly worked as a paraprobiotic (Tsilingiri and Rescigno, 2013), because *P. sabiae* did not colonize zebrafish guts (Supplementary Table S2).

### 4.3. The increased taurine concentration in the *Paraburkholderia sabiae*-exposed zebrafish brain

Metagenomic profiling using PICRUSt revealed functional alterations in the KEGG pathway involved in taurine metabolism (Figure 2B; Supplementary Table S7), and we demonstrated that taurine concentration was significantly (*p* < 0.05) increased in the *P. sabiae*-exposed zebrafish brain (Figure 3A). The presence of taurine in the colon depends on the gut microbiome, and it is transported from the colon into the systemic circulation (Matsumoto et al., 2017; Sharon et al., 2019; Li et al., 2020), including the central nervous system, through the blood–brain-barrier *via* the taurine transporter TauT (Benrabh et al., 1995). In addition, taurine is synthesized in the brain neurons and astrocytes (Vitvitsky et al., 2011). In this study, *P. sabiae* exposure upregulated the taurine synthesis gene, *cdo*, in zebrafish brains (Figure 3C), suggesting the existence of indirect mechanisms induced by *P. sabiae* ingredients, which activate taurine synthesis in the brain.

Taurine, a small organic compound, is a major constituent of bile that exists mainly in the large intestine. It possesses many biological functions, including osmoregulation, membrane stability, intracellular calcium metabolism, anti-oxidation, and anti-inflammation in various tissues (Marcinkiewicz and Kontny, 2014; Menzie et al., 2014). In vertebrates, taurine is thought to have anxiolytic properties in vertebrates (Zhang and Kim, 2007). For example, mouse administered 0.5 mmol/kg taurine for 7 days showed anxiolytic-like properties in the plus-maze test (Chen et al., 2004). The taurine-deficient mice without CSAD exhibited increased anxiety-like behavior in the plus-maze test, which was compensated by the oral treatment with 0.2% taurine (Park et al., 2019). In our results (Figure 3), taurine concentration was significantly (*p* < 0.05) higher in the *P. sabiae*-exposed zebrafish brains, suggesting that the increased level of taurine is the cause of reduced anxiety in the *P. sabiae*-exposed zebrafish. Consistent with our results, Mezzomo et al. reported that treatment with taurine (3.2 mmol/L) decreased anxiety-like behaviors induced by acute exposure to conspecific alarm substances in zebrafish (Mezzomo et al., 2016, 2019). Interestingly, Mezzomo et al. detected anxiolytic effects only in the light and dark test, not in the novel tank test as we did in the present study, in the case of acute taurine administration. In other words, the light and dark test may also provide greater improvement in the *P. sabiae* group.

Thus, additional research into the molecular mechanisms underlying the increase in the zebrafish gut microbiome diversity, along with the increase in taurine concentration in the zebrafish brain, following *P. sabiae* administration is required, and future studies will aim to explore the same.

## 5. Conclusion

We demonstrated that administering *P. sabiae* to adult zebrafish lowered their anxiety levels and improved the diversity of the gut microbiota in zebrafish. Functional analysis of PICRUSt2 predicted fluctuations in taurine metabolism in the gut microbiome, and our results demonstrated that *P. sabiae* treatment increased taurine concentration in the brain. These findings indicate that *P. sabiae* can reduce zebrafish anxiety *via* the gut-microbiota axis.

## Data availability statement

The data presented in the study are deposited in the DDBJ repository, accession number PRJDB15161.

## Ethics statement

Ethical review and approval was not required for the animal study because ethical approval from the local Institutional Animal Care and Use Committee was not sought, because Japanese law does not mandate the protection of zebrafish.

## Author contributions

SI and YS designed the study, wrote, and revised the manuscript. SI, RA, HF, KH, LZ, HN, and IM contributed to the acquisition, analysis, and interpretation of the data. All authors contributed to the article and approved the submitted version.

## Funding

This work was partially supported by the Japan Society for the Promotion of Science KAKENHI (grant numbers JP18K18218 and 21K14953). The funders had no role in the study design, data collection and interpretation, or decision to submit the work for publication.

## Conflict of interest

The authors declare that the research was conducted in the absence of any commercial or financial relationships that could be construed as a potential conflict of interest.

## Publisher’s note

All claims expressed in this article are solely those of the authors and do not necessarily represent those of their affiliated organizations, or those of the publisher, the editors and the reviewers. Any product that may be evaluated in this article, or claim that may be made by its manufacturer, is not guaranteed or endorsed by the publisher.

## Supplementary material

The Supplementary material for this article can be found online at: https://www.frontiersin.org/articles/10.3389/fmicb.2023.1079187/full#supplementary-material

## Footnotes

^1^ https://arriveguidelines.org/
