# Supplementary material for: Paraburkholderia sabiae administration alters zebrafish anxiety-like behavior via gut microbial taurine metabolism
Source: Front Microbiol. 2023 Feb 16;14:1079187. doi: 10.3389/fmicb.2023.1079187 (PMC9977788; doi:10.3389/fmicb.2023.1079187)
Supplement: Supplementary file 10 [file Data_Sheet_1.DOCX]

Supplementary Material

# Supplementary Methods

## Preparation of a bacterial cell suspension

*P. sabiae* DSM 23623 was purchased from the German Collection of Microorganisms (DSMZ). The bacterium was grown in R2A medium (0.5 g/L yeast extract, 0.5 g/L proteose peptone (Difco no. 3), 0.5 g/L casamino acids, 0.5 g/L glucose, 0.5 g/L soluble starch, 0.3 g/L Na-pyruvate, 0.3 g/L K_2_HPO_4_, 0.05 g/L MgSO_4_ x 7 H_2_O, pH 7.2) at 30 °C under aerobic conditions. Bacterial cells were collected by centrifugation at 10,000 × *g* for 5 min and suspended in phosphate-buffered saline (PBS) containing 12% glycerol. The bacterial suspension was divided into small aliquots and stored at −80 °C.

## Gut microbiome analysis in zebrafish

DNA was extracted from the zebrafish intestine using a Quick-DNA Fecal/Soil Microbe Miniprep Kit (Zymo Research, Irvine, CA, USA). The 16S rRNA V3/V4 region in the DNA was amplified by PCR using the mix primers, which included the Illumina sequencing adapter (underline), ACACTCTTTCCCTACACGACGCTCTTCCGATCTNNNNN CCT ACGGGNGGCWGCAG, and GTGACTGGAGTTCAGACGTGTGCTCTTCCGATCTNNNNN GACTACHVGGGTATCTAATCC (Klindworth et al., 2013). PCR products were purified using AMPure XP (Beckman Coulter, Brea, CA, USA). Library preparation and sequencing were performed using the MiSeq Reagent Kit v3 (Illumina, San Diego, CA, USA) at 2 × 300 bp.

Only the read sequences containing the primer sequences were extracted, using the fastx barcode splitter tool of the FASTX-Toolkit ver. 0.0.14. The primer sequences from the extracted reads were deleted using the FASTX-Toolkit Fastx trimer. Sequences with a quality value of less than 20 were subsequently removed, and sequences with a length of less than 40 bases and their paired sequences were discarded using Sickle ver. 1.33. Reads with a minimum overlap of 10 bases were combined using FLASH ver. 1.2.11.

After removing chimeric and noisy sequences using the dada2 plugin in Qiime2 ver. A total of 2021.2 (Callahan et al., 2016;Bolyen et al., 2019) representative sequences and OTU tables were output. The phylogenetic analysis was performed by comparing the obtained representative sequences with the 97% OTU of Greengene ver. 13_8 using the feature classifier, alignment, and phylogeny plug-ins (DeSantis et al., 2006). Diversity analysis was performed using the diversity plugin of Qiime2.

Using the representative sequences from Qiime2, functional composition tables based on the EC number, Kyoto Encyclopedia of Genes and Genomes (KEGG) ortholog, cluster of orthologous groups, and METACYC were generated with phylogenetic investigation of communities by reconstruction of unobserved states 2 (PICRUSt2) ver. 2.3.0 b (Douglas et al., 2020). Linear discriminant analysis effect size (LEfSe) ver. 1.0.8 was used to test whether any strains differed in relative abundance between the groups (Segata et al., 2011).

## Gene expression analysis in the zebrafish brains by QPCR

The zebrafish brains were homogenized by bead beating, and RNAs were extracted using TRIzol (Thermo Fisher Scientific, Waltham, MA, USA). RNAs was purified using an RNeasy Plus Mini Kit (Qiagen, Germantown, MD, USA). RNA concentrations and purities of the purified RNA solutions were evaluated using a BioSpectrometer (Eppendorf, Hamburg, Germany). cDNAs were synthesized from approximately 0.1 µg RNAs using ReverTra Ace qPCR RT Master Mix with gDNA Remover kit (Toyobo, Osaka, Japan) following the manufacturer’s protocol. QPCR was performed using a StepOnePlus Real-Time PCR System (Thermo Fisher Scientific). The primers used in this study are listed in **Table S1** (Borrelli et al., 2016;Davis et al., 2016). Each 20-μL reaction mixture included PowerUp SYBR Green Master Mix (Thermo Fisher Scientific), template cDNA, and 0.5 µM of each primer. Melt curves were analyzed to ensure the specificity of primer annealing and lack of primer secondary structure. The data were normalized to *ef1a*, *beta-actin*, or *l13a* gene expression levels (Borrelli et al., 2016;Davis et al., 2016;Liu et al., 2017). Measurements for each gene were performed in triplicates.

**References**

Bolyen, E., Rideout, J.R., Dillon, M.R., Bokulich, N., Abnet, C.C., Al-Ghalith, G.A., Alexander, H., Alm, E.J., Arumugam, M., Asnicar, F., Bai, Y., Bisanz, J.E., Bittinger, K., Brejnrod, A., Brislawn, C.J., Brown, C.T., Callahan, B.J., Caraballo-Rodriguez, A.M., Chase, J., Cope, E.K., Da Silva, R., Diener, C., Dorrestein, P.C., Douglas, G.M., Durall, D.M., Duvallet, C., Edwardson, C.F., Ernst, M., Estaki, M., Fouquier, J., Gauglitz, J.M., Gibbons, S.M., Gibson, D.L., Gonzalez, A., Gorlick, K., Guo, J.R., Hillmann, B., Holmes, S., Holste, H., Huttenhower, C., Huttley, G.A., Janssen, S., Jarmusch, A.K., Jiang, L.J., Kaehler, B.D., Bin Kang, K., Keefe, C.R., Keim, P., Kelley, S.T., Knights, D., Koester, I., Kosciolek, T., Kreps, J., Langille, M.G.I., Lee, J., Ley, R., Liu, Y.X., Loftfield, E., Lozupone, C., Maher, M., Marotz, C., Martin, B.D., Mcdonald, D., Mciver, L.J., Melnik, A.V., Metcalf, J.L., Morgan, S.C., Morton, J.T., Naimey, A.T., Navas-Molina, J.A., Nothias, L.F., Orchanian, S.B., Pearson, T., Peoples, S.L., Petras, D., Preuss, M.L., Pruesse, E., Rasmussen, L.B., Rivers, A., Robeson, M.S., Rosenthal, P., Segata, N., Shaffer, M., Shiffer, A., Sinha, R., Song, S.J., Spear, J.R., Swafford, A.D., Thompson, L.R., Torres, P.J., Trinh, P., Tripathi, A., Turnbaugh, P.J., Ul-Hasan, S., Vander Hooft, J.J.J., Vargas, F., Vazquez-Baeza, Y., Vogtmann, E., Von Hippel, M., Walters, W., et al. (2019). Reproducible, interactive, scalable and extensible microbiome data science using QIIME 2. *Nature Biotechnology* 37**,** 852-857.

Borrelli, L., Aceto, S., Agnisola, C., De Paolo, S., Dipineto, L., Stilling, R.M., Dinan, T.G., Cryan, J.F., Menna, L.F., and Fioretti, A. (2016). Probiotic modulation of the microbiota-gut-brain axis and behaviour in zebrafish. *Sci Rep* 6**,** 30046.

Callahan, B.J., Mcmurdie, P.J., Rosen, M.J., Han, A.W., Johnson, A.J., and Holmes, S.P. (2016). DADA2: High-resolution sample inference from Illumina amplicon data. *Nat Methods* 13**,** 581-583.

Davis, D.J., Doerr, H.M., Grzelak, A.K., Busi, S.B., Jasarevic, E., Ericsson, A.C., and Bryda, E.C. (2016). Lactobacillus plantarum attenuates anxiety-related behavior and protects against stress-induced dysbiosis in adult zebrafish. *Sci Rep* 6**,** 33726.

Desantis, T.Z., Hugenholtz, P., Larsen, N., Rojas, M., Brodie, E.L., Keller, K., Huber, T., Dalevi, D., Hu, P., and Andersen, G.L. (2006). Greengenes, a chimera-checked 16S rRNA gene database and workbench compatible with ARB. *Appl Environ Microbiol* 72**,** 5069-5072.

Douglas, G.M., Maffei, V.J., Zaneveld, J.R., Yurgel, S.N., Brown, J.R., Taylor, C.M., Huttenhower, C., and Langille, M.G.I. (2020). PICRUSt2 for prediction of metagenome functions. *Nat Biotechnol* 38**,** 685-688.

Klindworth, A., Pruesse, E., Schweer, T., Peplies, J., Quast, C., Horn, M., and Glockner, F.O. (2013). Evaluation of general 16S ribosomal RNA gene PCR primers for classical and next-generation sequencing-based diversity studies. *Nucleic Acids Res* 41**,** e1.

Liu, C.L., Watson, A.M., Place, A.R., and Jagus, R. (2017). Taurine Biosynthesis in a Fish Liver Cell Line (ZFL) Adapted to a Serum-Free Medium. *Mar Drugs* 15.

Segata, N., Izard, J., Waldron, L., Gevers, D., Miropolsky, L., Garrett, W.S., and Huttenhower, C. (2011). Metagenomic biomarker discovery and explanation. *Genome Biol* 12**,** R60.

# Supplementary Figures

##
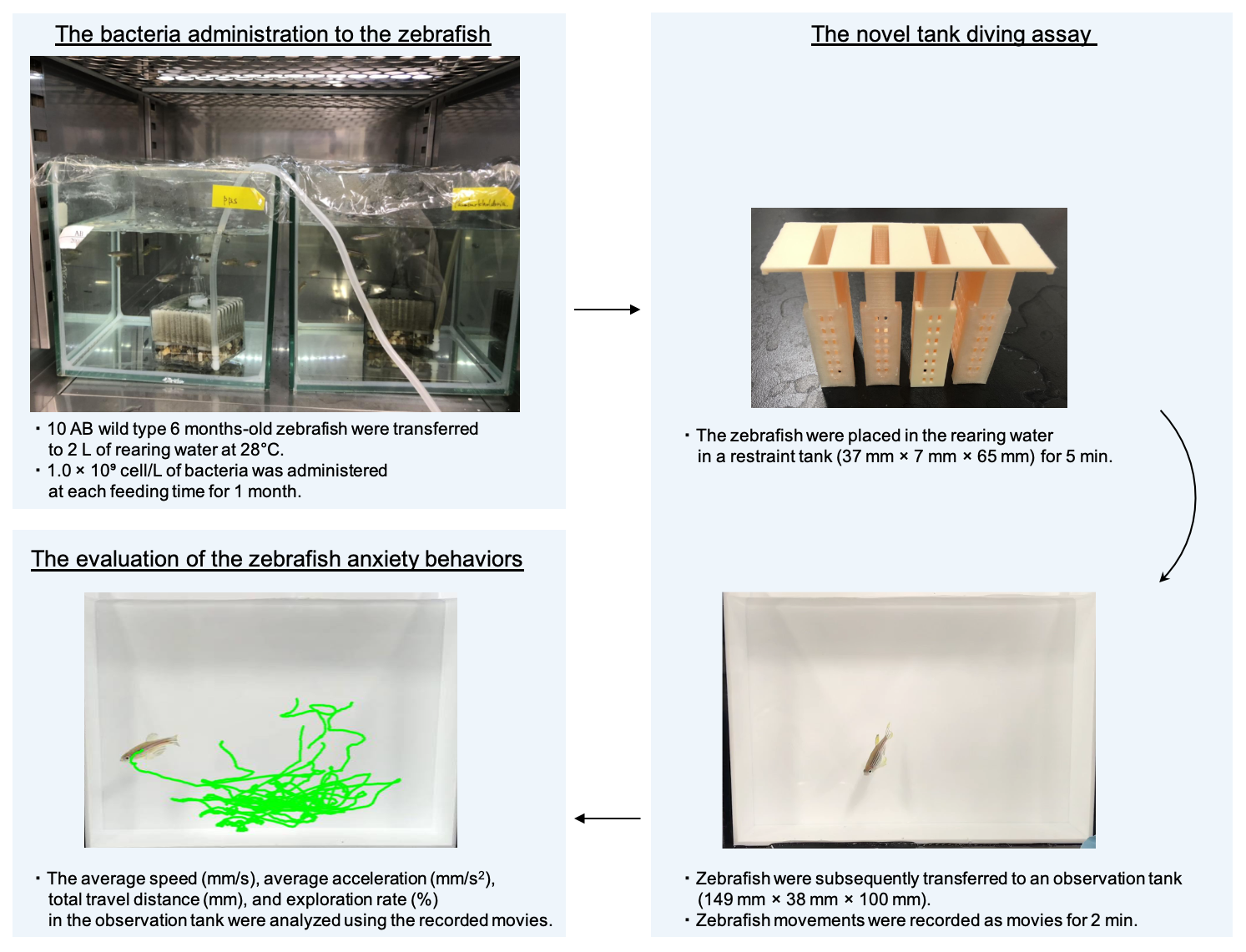


**Supplementary Figure 1.** The schematic image of the evaluation of the zebrafish anxiety-like behavior in this study

**
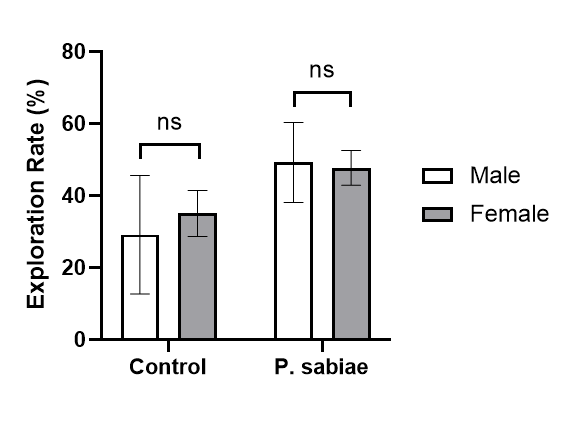
**

## Supplementary Figure 2. The zebrafish anxiety behaviors evaluated by the novel tank diving test. The exploration rate in the observation tank (%) of the zebrafish is presented. The error bars indicate standard deviation. N = 2-3. ns indicates not significant.


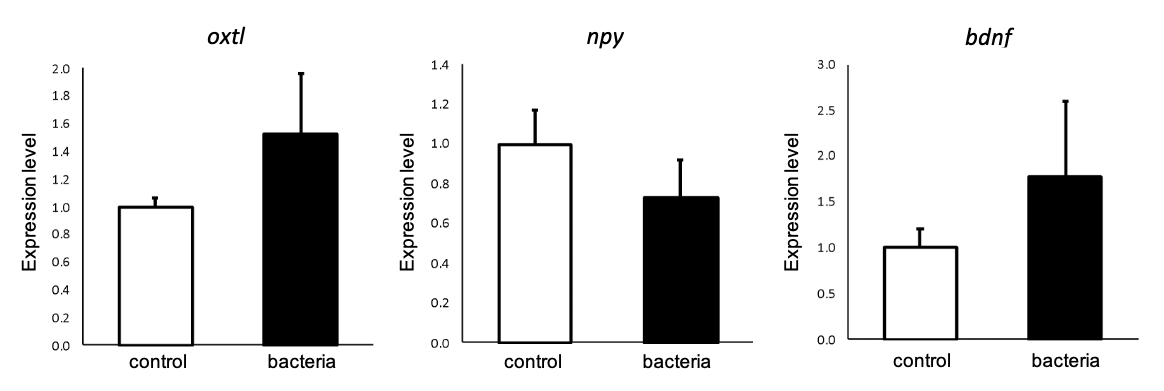


**Supplementary Figure 3.** The expression levels of neuropeptides and brain derived neurotrophic factor gene in zebrafish brains

The relative expression levels of neuropeptide synthesis genes, isotocin gene *oxtl*, neuropeptide Y gene *npy*, and brain derived neurotrophic factor gene *bdnf*, in the eight zebrafish brains were evaluated by QPCR. The data were normalized to *ef1a* or *beta-actin* gene expression levels. Error bars show standard error.


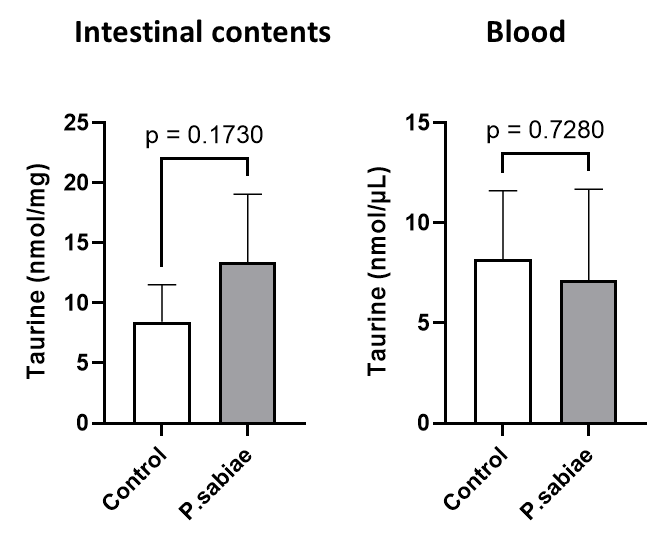


**Supplementary Figure 4.** Taurine levels in zebrafish intestinal contents and blood evaluated using the Taurine Assay Kit (Cell Biolabs.). N = 5.

**
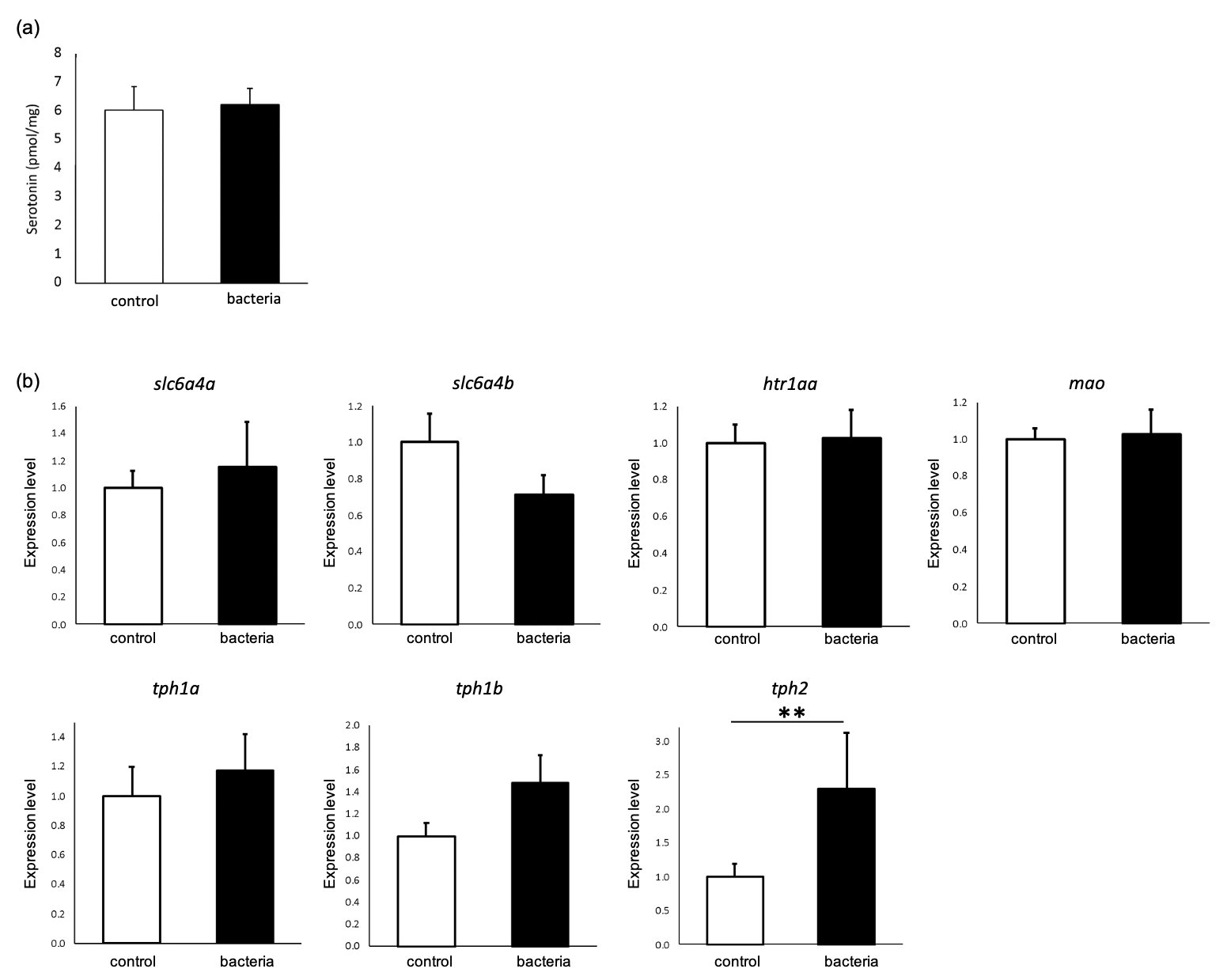
**

**Supplementary Figure 5.** Serotonin concentration and the expression levels of serotonergic genes in zebrafish brains

(a) Serotonin concentration in the zebrafish brains was evaluated using Serotonin ELISA Kit (Immusmol). N = 5. (b) The relative expression levels of serotonin transporter genes *slc4a6a* and *slc4a6b*, serotonin receptor gene *htr1aa*, *mao*, tryptophan hydroxylase genes *tph1a,* *tph1b,* and *tph2*, in the eight zebrafish brains were evaluated by QPCR. The data were normalized to *ef1a* or *beta-actin* gene expression levels. Error bars show standard error.


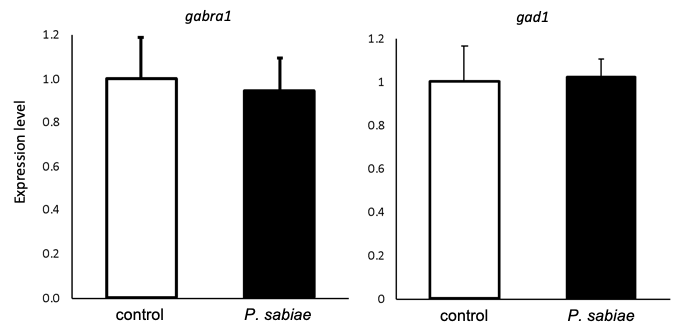


**Supplementary Figure 6.** The expression levels of GABAergic genes in zebrafish brains.

The relative expression levels of GABAergic genes, GABA-A alpha 1 receptor (*gabra1*) and glutamic acid decarboxylase (*gad1*) in the eight zebrafish brains were evaluated by QPCR. The data were normalized to *ef1a* gene expression levels. Error bars show standard error.
